# DASNeRF: depth consistency optimization, adaptive sampling, and hierarchical structural fusion for sparse view neural radiance fields

**DOI:** 10.1371/journal.pone.0321878

**Published:** 2025-05-12

**Authors:** Yongshuo Zhang, Guangyuan Zhang, Kefeng Li, Zhenfang Zhu, Peng Wang, Zhenfei Wang, Chen Fu, Xiaotong Li, Zhiming Fan, Yongpeng Zhao

**Affiliations:** 1 School of Information Science and Electrical Engineering, Shandong Jiaotong University, Jinan, Shandong, China; 2 Shandong Zhengyuan Yeda Environmental Technology Co., Ltd, Jinan, Shandong, China; Purdue University, UNITED STATES OF AMERICA

## Abstract

To address the challenges of significant detail loss in Neural Radiance Fields (NeRF) under sparse-view input conditions, this paper proposes the DASNeRF framework. DASNeRF aims to generate high-detail novel views from a limited number of input viewpoints. To address the limitations of few-shot NeRF, including insufficient depth information and detail loss, DASNeRF introduces accurate depth priors and employs a depth constraint strategy combining relative depth ordering fidelity regularization and depth structural consistency regularization. These methods ensure reconstruction accuracy even with sparse input views. The depth priors provide high-quality depth data through a more accurate monocular depth estimation model, enhancing the reconstruction capability and stability of the model. The depth ordering fidelity regularization guides the network to learn relative relationships using local depth ranking priors, reducing blurring caused by inaccurate depth estimation. Depth structural consistency regularization maintains global depth consistency by enforcing continuity across neighboring depth pixels. These depth constraint strategies enhance DASNeRF’s performance in complex scenes, making 3D reconstruction under sparse views more accurate and natural. In addition, we utilize a three-layer optimal sampling strategy, consisting of coarse sampling, optimized sampling, and fine sampling during the three-layer sampling process to better capture details in key regions. In the optimized sampling phase, the sampling point density in key regions is adaptively increased while reducing sampling in low-priority regions, enhancing detail capture accuracy. To alleviate overfitting, we proposed an MLP structure with per-layer input fusion. This design preserves the model’s detail perception ability while effectively avoids overfitting. Specifically, each layer’s input includes the output features from the previous layer and incorporates processed five-dimensional information, further enhancing fine detail reconstruction. Experimental results show that DASNeRF outperforms state-of-the-art methods on the LLFF and DTU dataset, achieving better performance in metrics such as PSNR, SSIM, and LPIPS. The reconstructed details and visual quality are significantly improved, demonstrating DASNeRF’s potential in 3D reconstruction under sparse-view conditions.

## Introduction

Novel view synthesis is a key research area in computer vision, aiming to reconstruct 3D scenes from multi-view 2D images and render images from any novel viewpoint. In-depth research across various fields enables the acquisition of accurate 3D information about objects, including virtual reality [1], autonomous driving [2], and localization and navigation [3], and face reconstruction [4], among others.

Traditional modeling methods rely on explicit representations that use geometric information to describe real-world scenes. Common geometric representations include textured meshes [5], voxels [6], and point clouds [7]. Although traditional modeling methods provide accurate 3D structural information, they are highly complex and heavily dependent on geometric models, making it challenging to represent complex and diverse scenes.

Implicit neural representations effectively address the limitations of explicit representations. 3D reconstruction algorithms based on implicit representations learn the nonlinear relationships between pixel values and 3D geometry, allowing for better handling of complex geometric transformations and scenes, such as perspective and occlusion. NeRF [8] can simultaneously learn the geometric structure and appearance information of a scene during training, allowing for flexible adjustments of viewpoint and lighting conditions during rendering. Leveraging these advantages, NeRF has elevated implicit reconstruction to a new level, producing stunning results in novel view synthesis of scenes. Although Neural Radiance Fields have revolutionized high-fidelity 3D scene reconstruction, they often require substantial computational resources and time. However, in many real-world scenarios, collecting densely sampled viewpoints is both expensive and time-consuming [9]. Therefore, developing few-shot NeRF methods that can learn from sparse viewpoints without significantly compromising performance is particularly important. In recent years, several approaches have significantly advanced sparse reconstruction in NeRF, falling into three main categories: The first category includes geometric constraint strategies [10–12], utilizing geometric sparsity, continuity regularization, and semantic information to enhance model performance with sparse inputs. The second category consists of pre-training-based strategies [13, 14], which enhance generalization by pre-training on similar scenes. For example, PixelNeRF [13] uses convolutional feature maps as conditional inputs, enabling effective inference of geometry and appearance in the target scene. The third category leverages depth maps [15–17] as supervision signals during training by employing linearly scaled depth maps to guide depth prediction in few-shot NeRF. However, for real-world depth maps, particularly those derived from pre-trained depth models or drone depth sensors, applying local region-based scale-invariant depth constraints, as in MonoNeRF [16], can be overly restrictive, especially when significant depth variations exist within the scene.

To enhance the clarity of complex details in few-shot reconstruction, we propose DASNeRF, a simple and effective solution based on the third category of methods. By optimizing the sampling strategy, enhancing the MLP network, and extracting depth priors from more accurate pre-trained monocular depth models [18] or coarse depth maps generated by consumer-grade depth sensors, we have achieved significant performance gains. Although depth maps are relatively easy to obtain in real-world scenarios, accurately extracting monocular depth estimates from pre-trained models can be challenging. Furthermore, inaccurate monocular depth estimates can cause inconsistencies during volumetric rendering, resulting in discrepancies between the expected depth and the actual geometric structure.

High-quality input images are essential for NeRF to produce high-quality results. In other words, training a NeRF model typically requires input images with high visibility, where nearly every pixel can accurately represent the scene’s lighting and object colors. In NeRF, input images are sampled to generate multiple points along each ray, which correspond to specific coordinates in 3D space. The five-dimensional information for each sampling point is then input into the subsequent MLP network, making the sampling process a crucial data input phase. During the sampling phase, to address the blurring of details caused by lighting changes and reduce the number of invalid sampling points, we did not use the standard coarse and fine sampling approach in NeRF. Instead, we restructured the sampling layer into three layers and introduced a dynamic optimal sampling strategy. By fully utilizing the training data and eliminating the interference from coarse depth maps and invalid sampling points, we improved reconstruction accuracy. Furthermore, the sampled points are processed before being fed into the MLP network. In the MLP, we employed an input fusion approach, where each layer’s input is a combination of the output features from the previous layer and the processed five-dimensional information, thereby enhancing the detail reconstruction of each sampled pixel.

DASNeRF provides a more scalable and precise solution for few-shot view synthesis. We evaluated the performance of DASNeRF on the LLFF dataset, testing its rendering quality and metrics such as PSNR, SSIM, and LPIPS. The results show that DASNeRF not only performs better across all metrics, but also achieves significantly higher rendering quality compared to our baseline method, demonstrating its superior capability in generating high-quality images with rich details. Our main contributions are as follows:

- We improve the accuracy of monocular depth estimation, enhancing the quality of coarse depth in input images and reducing the likelihood of inaccurate depth information
- Our dynamic optimal sampling strategy prioritizes effective sampling points, further enhancing the quality of detailed reconstruction.
- We incorporate an input fusion strategy into the MLP network to reduce overfitting and enhance the model’s ability to reconstruct fine details.

## Related work

### Neural radiance fields (NeRF)

NeRF demonstrates outstanding performance in novel view synthesis for complex scenes [19–23], leveraging its neural network-based implicit function representation to generate high-quality images from arbitrary viewpoints in 3D space . Many studies have proposed various improvements and extensions based on NeRF’s strong performance in novel view synthesis, 3D reconstruction, and dynamic scene generation. For example, CityNeRF [24], Mega-NeRF [25], UrbanNeRF [26], and MetaNeRF [27] extend the traditional NeRF model to city-scale or larger scenes. Meanwhile, GNeRF [28], SLAM-NeRF [29], and FlowNeRF [30] combine NeRF with other techniques to reduce the strict requirements on camera poses, enabling new view synthesis even without complete camera pose information.Additionally, Mip-NeRF [31] addresses aliasing by using cone tracing instead of traditional point tracing, while simplifying NeRF’s coarse and fine MLP networks into a single multi-scale MLP, enhancing the model’s robustness in dense view synthesis tasks.Similarly, some studies have further improved NeRF’s performance through anti-aliasing techniques and sparse 3D grids combined with spherical harmonics [32]. Other methods [33–35] have applied NeRF to dynamic scenes, extending its effectiveness and applicability across different use cases.

However, all of the aforementioned methods focus on scene reconstruction under dense view conditions. Therefore, training with sparse input images and synthesizing new high-quality images from unseen viewpoints remain challenges that are yet to be fully addressed in the novel view synthesis task.In existing research, few-shot NeRF [36–39] has gained increasing attention to improve model performance in sparse scenes, achieving accurate reconstruction from limited viewpoints through geometric and appearance regularization strategies [10, 15, 40, 41].In this paper, we focus on sparse-view NeRF to reduce the need for dense viewpoint capture in real-world applications.

### Few-shot novel view synthesis

Novel View Synthesis is a key research direction in computer vision, aiming to generate new viewpoints from existing multi-view or single-view images to achieve more realistic 3D scene rendering.In the realm of novel view synthesis for dynamic scenes, researchers have introduced a method for monocular novel view synthesis of dynamic scenes, addressing the challenge of reconstructing globally coherent depth from a moving camera. The proposed Depth Fusion Network integrates single-view depth and multi-view stereo depth to correct scale inconsistencies and produce a complete, view-invariant depth map. A DeepBlender network then synthesizes photorealistic novel views by blending dynamic foregrounds and static backgrounds. Evaluations on a real-world dataset show state-of-the-art performance in depth estimation and view synthesis. Despite limitations in extreme viewpoints and cluttered scenes, this approach significantly improves dynamic scene reconstruction [42].Furthermore, for novel view synthesis in Augmented Reality, researchers have proposed a new method for estimating the 3D vehicle pose from a single image, which is a key task in AR applications. The approach combines pre-trained semantic segmentation with enhanced single-image depth estimation to accurately determine the vehicle’s location. A key innovation is the pose estimation technique, which utilizes 2D projections of rotated point clouds to predict object rotation. This enables precise adjustments in the position, rotation, and scale of augmented objects, allowing virtual vehicles to be seamlessly placed in real-world scenes. Notably, the model achieves competitive performance without relying on ground truth 3D pose labels, highlighting its potential for real-world applications in augmented reality and autonomous driving [43]. The aforementioned studies demonstrate that integrating depth estimation, geometric consistency constraints, and self-supervised learning can significantly enhance the rendering quality and geometric fidelity of novel view synthesis, offering promising applications in areas such as dynamic scenes and augmented reality. However, although the methods discussed above can generate high-quality novel views under scenarios where multi-view or single-view depth priors are abundant, they face considerable challenges in the presence of sparse input. In particular, when only a very limited number of viewpoints are available, volumetric rendering approaches such as NeRF struggle to accurately capture 3D structures, often resulting in geometric distortions, view inconsistencies, and artifacts. Consequently, few-shot view synthesis has attracted increasing attention in recent years.

In recent years, few-shot NeRF methods can be broadly categorized into three types. The first type of method is based on geometric or semantic continuity constraints to improve the model’s performance with sparse inputs. For example, RegNeRF [10] imposes continuity constraints on the geometric structure, regularizing the appearance of image regions from unobserved viewpoints through a flow model. InfoNeRF [11], on the other hand, adopts ray entropy minimization regularization to encourage sparsity along ray directions, while using information gain reduction regularization to ensure depth continuity between neighboring rays. These methods impose continuity constraints on geometry and semantics, helping the model maintain consistency when generating novel viewpoints.

The second type of method attempts to pre-train the NeRF model on similar scenes and then fine-tune it on the target scene to enhance performance with few-shot inputs. PixelNeRF [13] processes image inputs using convolution, allowing the model to learn rich prior information from different scenes, thereby reducing the reliance on dense views. Similarly, MVSNeRF [14] combines plane-sweeping volumes with physics-based volumetric rendering, training first on other real scenes and then fine-tuning on the target scene. Such pre-training strategies enable the model to learn rich prior knowledge from multi-view data, improving its adaptability to new scenes. In addition, GeoNeRF [44] integrates cascaded cost volumes with a Transformer-based framework to achieve novel view synthesis without per-scene optimization. Its geometry reasoning module employs an FPN to extract 2D semantic features and leverages cost volumes to enhance depth estimation. The rendering module uses multi-head attention to fuse multi-view information and incorporates an autoencoder to optimize geometric consistency along the ray dimension, thereby reducing artifacts and improving detail. Nevertheless, GeoNeRF still has certain limitations. First, constructing the cascaded cost volumes entails considerable computational and storage overhead, affecting processing efficiency for high-resolution scenes. Second, although the Transformer-based multi-head attention mechanism improves geometric consistency, it also demands a large amount of GPU memory, which poses challenges for resource-constrained devices. Additionally, GeoNeRF is highly dependent on the quality of the geometric priors from source views; in scenarios with extremely sparse or uneven view distributions, its geometric reasoning may become inaccurate. Moreover, while the GeoNeRF+D variant leverages RGBD data to enhance depth perception, it is sensitive to the quality of the depth inputs, and significant noise can degrade rendering performance. Future work could optimize the cost-volume computation to reduce computational overhead, refine the Transformer architecture to lower memory usage, and bolster robustness under sparse-view conditions and low-quality depth inputs.

The third type of method uses depth information to supervise NeRF training. DSNeRF [15] utilizes sparse 3D points generated by COLMAP [45] or depth maps obtained from high-precision scanners to provide additional depth supervision. SparseNeRF [17] proposes a ranking-based depth regularization, extracting robust depth ordering from coarse depth maps obtained through pre-trained depth models or consumer-grade sensors. However, SparseNeRF’s monocular depth estimation lacks precision. Therefore, DASNeRF utilizes more accurate depth estimation along with corresponding global and local depth regularization to establish consistent spatial depth information from monocular depth estimation, allowing the model to generate more consistent scenes when handling sparse inputs. Additionally, in the cone tracing sampling of sparse views,DASNeRF employs a unique sampling approach to more precisely handle details, differing from recent sparse reconstruction methods for small scenes like RefNeRF [10], FreeNeRF [41], and SparseNeRF [17].

### Monocular depth estimation

Monocular depth estimation aims to infer scene depth information from a single 2D image, making it inherently challenging due to the absence of direct geometric cues. Nevertheless, it has substantial applications in areas such as autonomous driving, augmented reality, and robotics. In recent years, with the advancement of deep learning, monocular depth estimation methods have made significant progress.

Early monocular depth estimation methods primarily relied on handcrafted features and geometric priors. For instance, Saxena *et al*. [46] proposed a method based on Markov Random Fields (MRF) to infer depth by extracting features such as textures and edges from the image. Additionally, some methods utilized geometric priors of the scene, such as the ground plane assumption, to estimate depth [47]. However, these approaches often rely on strong assumptions, making them difficult to apply to complex scenes and limiting their accuracy.

With the rise of deep learning, monocular depth estimation methods have made significant progress. Eigen *et al*. [48] were the first to propose a convolutional neural network (CNN)-based framework for monocular depth estimation, using a multi-scale network to predict depth from a single image. Subsequent research further improved the network architecture and loss functions. For example, Laina *et al*. [49] proposed a deeper network structure and used the inverse Huber loss function to enhance the accuracy of depth predictions. However, traditional deep learning methods typically rely on large amounts of labeled data for training, which poses challenges such as high labeling costs and difficulty in data acquisition. To address these issues, an unsupervised learning framework was proposed for simultaneously learning camera pose regression and scene depth estimation from monocular video frames [50]. This method utilizes geometric consistency constraints within video sequences to achieve accurate depth estimation and pose prediction without any ground truth data, significantly enhancing the model’s generalization ability and robustness. Furthermore, researchers have proposed various self-supervised strategies to enhance the performance of monocular depth estimation. For example, an innovative framework combining spectral consistency and novel view synthesis has been introduced [51]. This method avoids incorrect matching in smooth regions through spectral consistency, while utilizing novel view synthesis algorithms to generate depth information from different camera perspectives. By designing confidence maps and fusion strategies, the disparity maps generated by the two methods are effectively combined, thereby improving depth estimation accuracy, particularly in correcting object boundaries and error-prone regions. Experimental results show that this method achieves outstanding performance across multiple datasets, demonstrating stronger robustness, especially when handling complex scenes.

In the task of single-image depth estimation, an increasing number of self-supervised learning methods have shown outstanding performance. Most existing self-supervised approaches rely on monocular video or stereo image pairs, constructing photometric and geometric consistency through forward and backward image transformations. However, these methods often overlook the impact of defocus blur on depth estimation, leading to lower depth estimation accuracy in defocused regions. To address this issue, researchers proposed an innovative self-supervised depth estimation framework that combines defocus and focus-based depth estimation methods (DfD and DfF) [52]. This framework enhances depth estimation accuracy by learning depth maps from the blur information in a single image and generating simulated focus-stack images and all-focus images.

In terms of generalization capability for monocular depth estimation, MiDaS [53] achieves zero-shot generalization across datasets through multi-dataset joint training and a scale-invariant loss function. This framework is capable of predicting high-quality relative depth maps from a single image and performs exceptionally well across various scenes and datasets, without relying on a specific depth range or absolute depth values. This approach has broad application potential in fields such as autonomous driving and augmented reality.Furthermore, DepthAnything [54] significantly enhances the generalization capability and robustness of monocular depth estimation by introducing large-scale unlabeled data and self-supervised learning strategies. This method leverages pre-trained vision foundation models (such as CLIP) to extract rich semantic features and optimizes depth prediction through multi-scale feature fusion and an adaptive loss function. Experimental results demonstrate that DepthAnything achieves outstanding performance on several public datasets, including KITTI, NYU Depth V2, and DIODE, with particularly strong results in cross-domain generalization and complex scene scenarios, outperforming existing methods. Additionally, DepthAnything showcases its potential for real-time applications, offering new possibilities for practical deployment in fields such as autonomous driving and augmented reality.

## Methods

**Fig 1:**
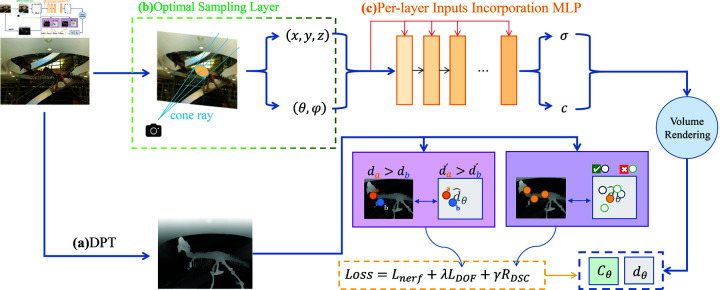
Overview of DASNeRF architecture. (**a**) We first use the DepthAnythingV2 model to obtain depth maps of the input views as a depth prior,Then, the depth information is used as a reference for regularizing the rendered depth information through the Depth Ordering Fidelity Loss and Depth Structural Consistency Regularization. The full objective loss of DASNeRF is formulated by $Loss=L_{nerf}+\lambda L_{DOF}+\gamma R_{DSC}$, where λ and γ control the importance of the regularization terms. In our model, we set λ=0.2 and γ=0.02 (**b**) We employ both 3D cone frustum sampling from Mip-NeRF and a three-layer dynamic sampling mechanism to obtain the five-dimensional information of the sampled points, which includes coordinates and ray information. (**c**) The sampled five-dimensional information serves as input, and we train using an MLP with per-layer input fusion. Each MLP layer takes two inputs: the features from the previous layer and the processed five-dimensional input information.After the MLP output, the color information *c* and volumetric density σ are obtained, and the color and depth information are rendered through volume rendering.

Our model presents an optimized Neural Radiance Field (NeRF) approach for sparse inputs, specifically aimed at improving sparse reconstruction details. First, to obtain accurate depth information for precise detail reconstruction, we use more precise monocular depth estimation to train depth maps, extracting useful depth priors to guide NeRF’s learning and applying robust relative depth regularization to these depth maps. Second, to reduce the impact of lighting on detail reconstruction, we introduce a dynamic optimal sampling layer that decomposes NeRF’s implicit radiance field and uses a enhanced NeRF model to monitor key regions in the scene, resulting in more accurate sampling. Additionally, to better reconstruct details matching the real scene, we merge each layer’s input with a five-dimensional vector in the MLP network to enhance detail preservation.The architecture of our training process is shown in Fig 1 .

### Background: neural radiance fields

NeRF [8], unlike 3D meshes, point clouds, and voxels, introduces implicit functions to model 3D scenes and uses volumetric rendering to synthesize images. Compared to voxel-based representation methods, NeRF overcomes the limitations of resolution and storage space, enabling the synthesis of high-quality results. It uses a neural network to map 3D positions *x* and viewing directions *d* to density σ and color *c* for image rendering (σ,c)=f(γ(x),γ(d)),where *f* is a mapping function typically composed of a multi-layer perceptron(MLP), and γ is position encoding.

Then, we render each expected pixel color $\hat{C}(r)$ by projecting rays $r\left(t\right)=o+t_{d}$ with near boundaries *t*_*n*_ and far boundaries *t*_*f*_.The method involves uniformly dividing the ray *r* along $\left[\begin{array}{c} t_{n},t_{f} \end{array}\right]$ into N points $\left(\begin{array}{c} t_{1},\;t_{2},\;...,t_{n} \end{array}\right)$, and calculating the pixel color $\hat{C}(r)$ using Eq 1.

$$\hat{C}(r)=\sum_{i=1}^{N}\omega_{i}c_{i},$$ (1)

$$\omega_{i}=T_{i}\alpha_{i},$$ (2)

$$T_{i}=exp\left(-\sum_{j=1}^{i-1}\sigma_{j}\delta_{j}\right),$$ (3)

$$\alpha_{i}=1-exp(-\sigma_{i}\delta_{i})$$ (4)

Where $\delta_{i}$ represents the interval between samples along the ray *r*. The training objective of NeRF, *L*_*nerf*_, is the mean squared error between the actual pixel color *C*(*r*) and the rendered pixel color $\hat{C}(r)$:

$$L_{nerf}=\sum_{r\in R}\|C(r)-\hat{C}(r){\|}_{2}^{2}$$ (5)

where *R* is the set of all rays from the camera center to the image pixels.

### More advanced depth priors

Based on our research, we found that the original Neural Radiance Fields (NeRF) typically require hundreds of images as input to render high-fidelity scenes. Although this method ultimately results in a good 3D representation, it significantly limits its practical use. Therefore, we aimed to develop a sparse reconstruction model that reduces the number of training views as much as possible. However, NeRF still faces significant challenges in sparse-view scenarios, mainly due to distortion and severe image artifacts caused by insufficient data, which are particularly evident in regions with extremely sparse points. To improve the quality of sparse reconstruction, we leverage pre-trained priors to obtain depth estimates from the training views, applying them during training. This allows for more accurate depth prediction and clearer image reconstruction.

Currently, most NeRF-based sparse view reconstruction methods that leverage pre-trained priors use MiDaS to obtain depth information. However, experiments have shown that MiDaS, as a monocular depth estimation method, faces limitations when handling complex scenes. It struggles to fully capture depth details, especially in scenarios involving transparent or reflective objects and intricate geometries, making it prone to noise and depth discontinuities. Additionally, single-view depth maps often come with rough annotations, and the imperfections of the depth estimation model, coupled with dataset biases, make accurate 3D depth estimation under monocular conditions extremely challenging. These factors often lead to uncertainties in depth estimation, thereby affecting the quality and stability of 3D reconstruction.

**Fig 2:**
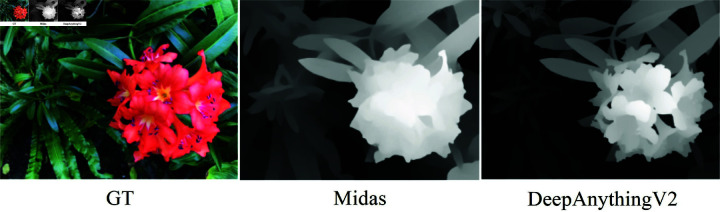
Depth map comparison of the two models. We compared the Midas and DepthAnything models on the same scene to generate a monocular depth map.

To address these challenges and improve the accuracy and robustness of depth estimation, we selected DepthAnythingV2, the latest depth estimation model. DepthAnythingV2 employs a DPT architecture with a DINOv2 backbone and is trained on around 600,000 synthetic labeled images and 62 million real unlabeled images, achieving state-of-the-art results in both relative and absolute depth estimation. Compared to MiDaS, DepthAnythingV2 excels in handling transparent, reflective, and complex geometric objects, reducing noise and providing more stable depth estimates.The comparison results on our model are shown in Fig 2. Additionally, DepthAnythingV2 provides an inference speed 10 times faster than MiDaS, making it highly suitable for applications requiring rapid depth map generation. Furthermore, DepthAnythingV2 is 10 times faster in inference compared to MiDaS, making it ideal for applications that require rapid depth map generation. We also compared DepthAnything V1 and V2. V1, as an initial exploration, used only the features from the last four layers of DINOv2 for decoding. In contrast, V2 incorporates intermediate features and includes comprehensive optimizations in both performance and interaction. This version emphasizes improved generalization, real-time processing, and support for multi-modal inputs, making it more suitable for large-scale and diverse application scenarios.Since our model relies on depth information, we first use an accurate monocular depth estimation model to provide high-precision depth data for the input images. This enables us to apply more effective constraints during the regularization of true and rendered depth.

Our model provides accurate depth information and introduces depth-related loss terms in the regularization process to further enhance reconstruction quality. Given the inherent coarseness of monocular depth estimation, we designed a relative depth ordering fidelity regularization along with a depth structural consistency strategy to ensure greater accuracy and consistency in the reconstruction.

#### Depth ordering fidelity loss.

In 3D reconstruction with limited training views, directly using coarse monocular depth estimation can result in blurring issues during sparse view reconstruction due to noise and inaccuracies in depth annotations. To overcome this challenge, we leverage a more accurate depth prior model and introduce relative depth ordering fidelity regularization, effectively mitigating these problems.

**Fig 3:**
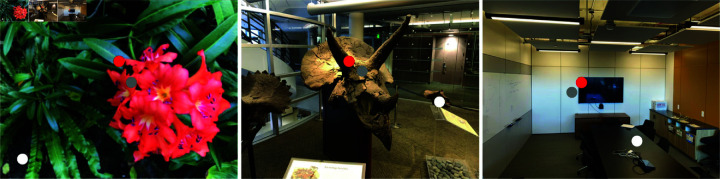
Depth ordering challenge in distant points. The depth estimation model can clearly distinguish that the green point is nearer than the gray point, but has difficulty comparing the red and gray points. For better clarity, please zoom in.

As shown in Fig 3, in an image, depth estimation models can easily determine the depth ordering of two nearby points, such as the red and gray points, but have difficulty estimating the depth ordering of two distant points, such as the red and white points. This indicates that depth ordering becomes less reliable as the spatial distance between points increases.

Therefore, we propose a relative depth ordering fidelity regularization method that extracts depth ordering priors from depth maps to guide the neural network. On one hand, given a local region *P* of an RGB image *I* and its corresponding local pose *p*,we calculate the depth of the ray originating *d*_*r*_ from *P* using Equation $d_{r}=\sum_{i=1}^{N}\omega_{i}t_{i}$. On the other hand, we use the pre-trained DepthAnythingV2 model to estimate the depth of image *I*,then crop a local depth map $d_{V2}$ that corresponds to the same spatial location as *d*_*r*_.We subsequently transfer the depth ordering information from $d_{V2}$ to *d*_*r*_.completing the relative depth ordering fidelity regularization. Let *k*_1_ and *k*_2_ represent the 2D pixel coordinates in $d_{V2}$ and *d*_*r*_,respectively; the depth ordering regularization can then be expressed as:

$$L_{DOF}=\sum_{d_{V2}^{k1}\leq d_{V2}^{k2}}max(d_{r}^{k1}-d_{r}^{k2}+mar,0)$$ (6)

Here,$d_{V2}^{k_{1}}$ and $d_{V2}^{k_{2}}$ represent the depth values of two pixels estimated from the pre-trained depth model, while *k*_1_ and *k*_2_ are the 2D coordinate indices of these two pixels. Additionally, $d_{V2}^{k_{1}}\leq d_{V2}^{k_{2}}$ indicates that the depth value of pixel *k*_1_ in the depth map is less than or equal to the depth value of pixel *k*_2_. $d_{r}^{k_{1}}$ and $d_{r}^{k_{2}}$ are the corresponding depth values of the predicted pixels.In Eq 6, $max(d_{r}^{k1}-d_{r}^{k2}+mar,0)$ is used to penalize the inconsistency between the depth ordering predicted by our model and the depth ordering from the pre-trained depth model. That is, if the depth ordering predicted by our model differs from the pre-trained model’s depth ordering (i.e.,$d_{V2}^{k_{1}}>d_{V2}^{k_{2}}$), a positive penalty term is generated. Here, mar is an adjustment of the boundary value. To enhance the adaptability of the depth regularization, we propose a dynamic boundary adjustment strategy. By dynamically calculating the value of mar, the model can adaptively adjust according to the depth differences of different pixels, effectively accommodating scenes with varying depth levels. The dynamic boundary adjustment formula is as follows:

$$mar=\alpha \cdot \frac{1}{N}\sum_{i=1}^{N}|d_{r}^{k1}[i]-d_{r}^{k2}[i]|$$ (7)

Here, α is a tunable hyperparameter used to control the magnitude of the margin, while N represents the number of depth samples in the computation area. $d_{r}^{k1}[i]$ and $d_{r}^{k2}[i]$ denote the depth values of the i-th pixel at different sampling layers, *k*_1_ and *k*_2_. Formula $\left|d_{r}^{k1}[i]\;-\;d_{r}^{k2}[i]\right|$ calculates the depth difference between different layers and dynamically determines the size of the margin through the mean value, allowing it to adapt to the depth characteristics of various scenes.We process the data obtained from the depth model using relative inverse depth. This dynamic boundary adjustment strategy provides more precise control over depth errors, effectively mitigating blurring issues caused by inaccurate depth estimates. Furthermore, dynamic adjustment allows the model to be more flexible and adaptive when dealing with complex depth variations, thereby enhancing its robustness and accuracy in sparse view reconstruction.

#### Depth structural consistency regularization.

The relative depth ordering fidelity regularization ensures that the depth map predicted by NeRF is consistent with the depth map generated by DepthAnythingV2. However, this constraint only addresses the depth of neighboring points in the image and cannot guarantee spatial continuity across the depth map. To solve this issue, we extract spatial continuity priors from the DepthAnythingV2 model to support scenarios involving large displacements between multiple depth pixels. If neighboring depth pixels in the DepthAnythingV2 depth map exhibit continuity, we apply the same continuity constraints to the corresponding depth pixels in NeRF. The depth structural consistency regularization formula is as follows:

$$R_{DSC}=\sum_{k1}\sum_{d_{V2}^{k2}\in KNN\left(d_{V2}^{k2}\right)}max(\left|d_{r}^{k1}-d_{r}^{k2}\right|-mar^{\prime },0)$$ (8)

Here, KNN(·) returns the k-nearest neighbors based on depth values within a small region. A small boundary value is used to account for minor depth variations between neighboring pixels.

Using the latest depth model helps standardize depth regularization, and, combined with more effective regularization strategies, clarifies the depth information of the scene. This allows for more precise reconstruction of both neighboring and distant pixel depths, ultimately enhancing the overall quality of scene reconstruction.

### Dynamic optimal sampling layer

In sparse-view 3D reconstruction, the limited number of training views often results in a loss of scene details, particularly when the number of views is low and sampling point quality is uncertain.This increases the likelihood of losing reconstruction details in regions with intricate features or complex depth structures. Consequently, 3D structural consistency across multiple viewpoints may be compromised, and rendering quality may become unstable.Furthermore, in scenes with specular reflections or low lighting, variations in illumination can affect the realism of the reconstruction.

To address these challenges, we propose a three-stage sampling strategy that introduces an optimal sampling layer between coarse and fine sampling.This layer adaptively adjusts the number and locations of new sample points based on their priorities from the previous stage, targeting high-weight regions to better capture subtle variations.By incorporating a dilation operation, we adaptively adjust sampling density in key areas, thereby enhancing sampling efficiency and ensuring rendering quality.

DASNeRF employs a three-stage sampling approach—coarse sampling, optimal sampling, and fine sampling—to better meet the requirements of sparse reconstruction. In the initial coarse sampling phase, we adopt conical rays, as used in Mip-NeRF [31], instead of traditional linear rays.We stratify sampling along the depth direction, distributing a few sample points from near to far planes to ensure initial coverage of the entire depth range.Building on this, the coarse sampling process introduces a moderate random offset δ in each interval to achieve a more uniform initial distribution, with δ randomly generated within the [0,1) range.

After completing the coarse sampling, we optimize the process based on the sampling priorities from the previous layer. The core advantage of this optimal sampling layer is its ability to dynamically determine the number of sampling points in each region based on priority, concentrating the sampling points in high-density and high-priority areas. Specifically, we introduced mechanisms such as dynamic sampling allocation, weight smoothing factors, and dynamic padding parameters to adaptively adjust sampling density across different regions. Furthermore, to extend the coverage of sampling points along the depth direction and more accurately capture critical details, we incorporated a dilation operation at this stage.In the optimal sampling region, dynamic sampling allocation can appropriately increase sampling density in high-priority areas. Compared to uniform sampling, our adaptive adjustment of the number of samples effectively reduces computations in low-priority areas, thus saving computational resources. To prevent over-concentration in certain areas during sampling, which could lead to overfitting, we introduced a weight smoothing factor to ensure smoother weight distribution and maintain a relatively balanced sampling pattern. Combined with dynamic padding parameters, this approach also helps to prevent detail loss. To further enhance accuracy, we introduced a dilation operation in the optimal sampling stage of the three-layer sampling process. During each sampling iteration, the sampling points in high-priority regions are expanded along the depth direction by a specified ratio, resulting in a broader sampling range within the depth space. This expansion not only increases sampling diversity but also improves coverage, significantly reducing the likelihood of missing subtle variations in the scene.

Finally, after the optimal sampling layer, we use the fine sampling layer from the original Mip-NeRF [31], using the dilated sampling points as the final input for the fine sampling stage. These points are further refined based on their weights along the ray. This fine sampling process allows the model to better capture local details by refining the sampling locations.

The three-layer sampling optimization, combined with the optimal sampling layer, makes the entire sampling process more flexible and precise. The optimal sampling layer performs weighted allocation based on the priority distribution from the coarse sampling stage, while the final fine sampling layer further refines the sampling according to the priorities provided by the optimal sampling. This three-layer approach effectively captures more details and accurately represents geometric features within the image. The hierarchical sampling design in DASNeRF significantly enhances reconstruction performance under sparse-view conditions, improving overall reconstruction accuracy.

### Per-layer inputs incorporation

In few-shot view synthesis, overfitting can be a significant issue. The most straightforward solution is to reduce the model parameters, such as decreasing the number of MLP layers, which can help alleviate overfitting to some extent, as proposed by DietNeRF [55]. However, this simplified NeRF often struggles to recover fine details, resulting in blurred novel views. To address these challenges, we propose an improved MLP network designed to mitigate overfitting. Our MLP network employs a multi-input structure, incorporating both 3D positions and 2D viewing directions at each layer input.Representing the five-dimensional information (x,y,z,θ,φ) as X,we need to convert the input five-dimensional coordinates into an encoded input embedding $\gamma_{L}(X)$. The input fusion formula can then be expressed as:

$$f_{i}=\phi_{i}(f_{i-1},\gamma_{L}(X)),f_{1}=\phi_{1}(\gamma_{L}(X))$$ (9)

where $\phi_{1}$ represents the MLP of the *i* ayer, and *f*_*i*_ is the corresponding layer’s output feature. Our method ensures that each layer of the MLP explicitly perceives the input embedding. This design allows the MLP at different depths to learn the output mapping from the input embedding.

In summary, our proposed multi-input MLP network outperforms the traditional NeRF model by effectively mitigating overfitting while maintaining a high level of detail preservation. By combining 3D position and 2D viewing direction for input fusion, our approach allows each layer of the MLP to explicitly perceive the five-dimensional input embedding, leading to more accurate view synthesis. Experimental results demonstrate that our method significantly surpasses traditional NeRF in few-shot view scenarios, not only in preventing overfitting but also in enhancing the detail fidelity of novel view synthesis.

## Experiments

### Datasets

We conduct experiments on the LLFF [56] and DTU [57] datasets. The LLFF dataset contains eight complex forward scenes. Following the setup in RegNeRF [10], we used every 8th image as the hold-out test set and evenly selected three views from the remaining images for training. Unlike LLFF, DTU is an object-level dataset. Following PixelNeRF [13], we use the same 15 scenes in our experiments, and similarly, 3 views are used for training. On the DTU dataset, the background of these scenes consists of a white table or a black background, with minimal texture. We used the monocular depth estimator DepthAnythingV2 [54] to derive depth priors. To avoid background bias during inference, we masked the background, as suggested in RegNeRF. To ensure fair comparison, we followed the same evaluation protocol.

### Evaluation metrics

In our experiments, we used three metrics commonly employed in NeRF evaluations: Peak Signal-to-Noise Ratio(PSNR), Structural Similarity Index (SSIM) (higher is better), and Learned Perceptual Image Patch Similarity (LPIPS) (lower is better) to quantitatively assess the reconstruction quality of different models.To ensure fairness, we trained all models on the same machine or server whenever possible. It is important to note that the NeRF model trained with dense views was used only to generate evaluation benchmarks and not as part of the model training process itself.

### Implementation details

We implemented DASNeRF based on the official JAX [58] implementation. The training process used the Adam optimizer to drive DASNeRF, with an exponentially decaying learning rate starting at $2\times 10^{-3}$ and gradually decreasing to $2\times 10^{-5}$. The initial batch size was set to 4096. We utilized the pre-trained DepthAnythingV2 model to generate monocular depth maps from the training views. Each scene was trained and inferred using a 24GB NVIDIA RTX 3090 on the AutoDL platform. Additionally, we implemented DASNeRF on a 16GB NVIDIA RTX 5000 and a 16GB NVIDIA RTX 2080 Ti.

### Comparisons on LLFF

In DASNeRF, for each input image, we assume that the camera parameters are known, as in applications such as robotics or augmented reality, external sensors or pre-calibrated camera sets can provide the camera poses.

Our model builds upon SparseNeRF with targeted improvements and optimizations, making our primary goal on the LLFF dataset a direct comparison with SparseNeRF to evaluate the effectiveness and enhancements in performance. Additionally, we conducted a comprehensive comparison of DASNeRF with several state-of-the-art methods for sparse view reconstruction, including PixelNeRF [13], MvsNeRF [14], Mip-NeRF [31], and RegNeRF [10]. Each of these methods excels in handling sparse view scenarios. By comparing them, we aim to comprehensively showcase the improvements in reconstruction quality and detail fidelity of DASNeRF under sparse input conditions.

We conducted a comparison focusing on accuracy, with the results summarized in Table 1. In our experiments, we categorized the comparative trials into three distinct groups. The first group included SRF, PixelNeRF, and MVSNeRF, which were pre-trained on similar scenes to acquire advanced semantic features and then directly tested on each scene from the LLFF dataset. In contrast, our model was fine-tuned on each specific scene of the LLFF dataset to better adapt to the unique characteristics of the data. The second group comprised Mip-NeRF, DietNeRF, and RegNeRF. Notably, Mip-NeRF was the first NeRF model specifically designed for dense view training, utilizing cone-shaped ray sampling, while all three models employed geometric continuity and semantic constraints during training to enhance performance.The third group of methods leveraged knowledge derived from depth maps or sparse points obtained from COLMAP, including DSNeRF, FreeNeRF, SparseNeRF, and the 2024 ColNeRF model.

**Table 1:** Quantitative Comparison on LLFF.Our proposed method outperforms other methods on real-world forward-facing scenes, ft indicates the results fine-tuned on each scene individually.

Method	PSNR(↑)	SSIM(↑)	LPIPS(↓)
SRF ft [37] (CVPR2021)	17.07	0.436	0.529
PixelNeRF ft [13] (CVPR2021)	16.17	0.438	0.512
MVSNeRF ft [14] (ICCV2021)	17.88	0.584	0.327
Mip-NeRF [31] (ICCV2021)	14.62	0.351	0.495
DietNeRF [55] (ICCV2021)	14.94	0.370	0.496
RegNeRF [10] (CVPR2022)	19.08	0.587	0.336
DSNeRF [15] (CVPR2022)	18.94	0.584	0.362
FreeNeRF [41] (CVPR2023)	19.82	0.638	0.336
SparseNeRF [17] (ICCV2023)	19.86	0.624	0.328
ColNeRF [59] (AAAI2024)	20.14	0.587	0.447
DASNeRF (our)	20.42	0.657	0.312

Our results indicate that, within the group employing depth maps or pre-extracted knowledge, our model consistently outperformed others. Furthermore, across all three groups, our model demonstrated a substantial advantage in reconstruction accuracy, achieving the highest scores on all three evaluation metrics.

**Fig 4:**
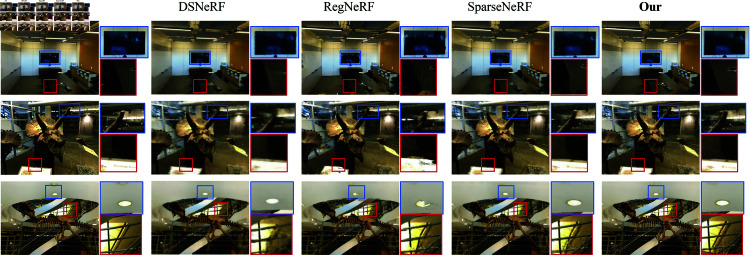
Visual comparisons on the LLFF dataset with three views. Red and blue boxes denote compared regions.DASNeRF achieves consistent improvement in different scenes.

We performed a qualitative analysis on the LLFF dataset, as shown in Fig 4, which displays the outputs of each method, the ground truth (GT), and the results of our model. In the figure, each row represents a different scene, with key details emphasized to enhance visual comparison. Our model clearly outperforms the baseline, SparseNeRF, in reconstruction quality, achieving sharper and more detailed results. We compared several state-of-the-art sparse view reconstruction methods, including RegNeRF, DSNeRF, and SparseNeRF. The results show that our model consistently outperforms these methods across all aspects, particularly excelling in pixel-level detail clarity.

The images clearly show that while all three methods extract information from depth maps or sparse points obtained from COLMAP, there are noticeable differences in pixel-level clarity. Our rendered scenes, in comparison, exhibit richer details, sharper edges, and more precise rendering of intricate features. Our model demonstrates superior fidelity in detail reconstruction compared to RegNeRF and DSNeRF. For example, in the room scene (first row), our model provides a clearer and more accurate reconstruction of the television screen and surrounding objects.

Our model also demonstrates significant improvements in depth estimation and lighting consistency compared to SparseNeRF. In the highlighted areas of room scene, our model captures lighting and depth variations more realistically, resulting in a more natural reconstruction. In scenes with complex textures, such as the horns scene (second row), our model excels at preserving details, with the contours and textures of the skull clearly visible, while competing methods like DSNeRF and SparseNeRF exhibit significant blurring and loss of definition in the same regions.

A common challenge for sparse-view NeRF models is the appearance of artifacts in areas with significant depth or light intensity variations. In the trex scene (third row), our model effectively reduces artifacts.For instance, in the ceiling lights and surrounding structures, our model captures fewer distortions and more accurately restores the underlying shapes compared to RegNeRF and DSNeRF, which exhibit noticeable inconsistencies in the same areas. These results confirm that DASNeRF can effectively model complex scenes with high fidelity.

### Comparisons on DTU

Similarly to the LLFF dataset, we again divide the comparison models under sparse input conditions into three different groups, as shown in Table 2. The first group includes SRF [37], PixelNeRF [13], and MVSNeRF [14], which are pre-trained on other DTU scenes and then fine-tuned on our selected 15 target scenes for testing.In the second group, to obtain pre-trained models, we partition the DTU dataset into a training set of 88 scenes and a test set of 15 scenes. However, because the training and test sets share similar scenes, the test scenes for Mip-NeRF [31], DietNeRF [55], and RegNeRF [10] in this group have a distribution similar to the training scenes. This setup enables these three models to achieve significantly more promising results.In the final third group, we compare the models DSNeRF [15], FreeNeRF [41], SparseNeRF [17], and ColNeRF [59], all of which leverage depth or priors derived from sparse points obtained via COLMAP.Our comparison results on the DTU dataset indicate that we not only achieve superior performance in the depth-prior-based sparse reconstruction category, but also obtain promising results in the first and second categories without needing pre-training on other scenes.

**Table 2:** Quantitative Comparison on DTU. Compared with other methods, our approach achieves better performance on the first two metrics in real-world forward-facing scenes. Here, ’ft’ denotes the results obtained by fine-tuning on each individual scene.

Method	PSNR(↑)	SSIM(↑)	LPIPS(↓)
SRF ft [37] (CVPR2021)	15.68	0.698	0.281
PixelNeRF ft [13] (CVPR2021)	18.95	0.710	0.269
MVSNeRF ft [14] (ICCV2021)	18.54	0.769	0.197
Mip-NeRF [31] (ICCV2021)	8.68	0.698	0.281
DietNeRF [55] (ICCV2021)	11.85	0.633	0.314
RegNeRF [10] (CVPR2022)	18.89	0.745	0.109
DSNeRF [15] (CVPR2022)	16.90	0.570	0.450
FreeNeRF [41] (CVPR2023)	19.92	0.736	0.324
SparseNeRF [17] (ICCV2023)	19.41	0.769	0.201
ColNeRF [59] (AAAI2024)	19.55	0.716	0.362
DASNeRF (our)	20.29	0.771	0.199

**Fig 5:**
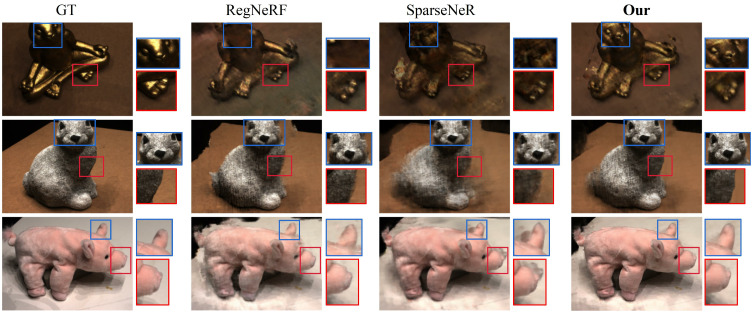
Visual comparisons on the DTU dataset with three views. Red and blue boxes denote compared regions.DASNeRF achieves consistent improvement in different scenes.

We also conducted a qualitative analysis on the DTU dataset, as illustrated in Fig 5. The figure compares the results of the Ground Truth (GT), RegNeRF [10], SparseNeRF [17], and our model. Each row corresponds to a different scene, and within each scene we highlight key details to enhance visual contrast. For instance, the first row of the figure shows scene 110 from the DTU dataset. We focus on the subject’s head and feet, demonstrating that our method excels in preserving fine details.In the third row (scene 103), our model demonstrates reduced artifacts around the subject’s nose and ears. As can be seen from the figure, DASNeRF outperforms the reference model SparseNeRF on the DTU dataset.

### Ablation studies

To demonstrate the effectiveness of our design choices, we performed ablation studies. In the ablation experiments, we systematically evaluated the contributions of the proposed More Advanced Depth Priors (DPT(V2)), Dynamic Optimal Sampling Layer (DOSL), and Per-Layer Inputs Incorporation (PII) to model performance. By incrementally adding or disabling these modules, we observed changes in Peak Signal-to-Noise Ratio (PSNR), Structural Similarity Index (SSIM), and Learned Perceptual Image Patch Similarity (LPIPS), allowing us to analyze the independent contributions and synergies of each module.

As shown in Table 3, the experimental results indicate that each of the three modules contributes differently to model performance improvement under sparse view conditions. Incorporating the advanced depth estimation module significantly improved depth estimation accuracy under sparse input conditions, reducing geometric distortion and depth errors.For instance, enabling only DPT(V2) increased PSNR to 20.14, SSIM to 0.631, and decreased LPIPS to 0.321, demonstrating that high-quality depth priors effectively optimize geometric consistency in reconstruction. The Dynamic Optimal Sampling Layer (DOSL) module improved the reconstruction quality of scene details by dynamically adjusting sampling density and incorporating dilation operations, resulting in denser sampling in high-weight areas. For instance, with DOSL enabled, PSNR increased to 20.05, SSIM rose to 0.627, and LPIPS decreased to 0.323.These results indicate that the dynamic sampling strategy significantly enhanced detail capture. The Per-Layer Inputs Incorporation (PII) module explicitly combines 3D positional information with 2D viewing direction, improving detail perception and effectively mitigating overfitting under sparse view conditions. With only PII enabled, PSNR increased to 20.08, SSIM reached 0.630, and LPIPS decreased to 0.323, indicating that this module enhanced the model’s ability to preserve details and resist overfitting.

**Table 3:** Ablation studies. We perform ablation studies on LLFF with 3 input views, where DPT(V2) means more advanced depth priors, DOSL means dynamic optimal sampling layer and PII means per-Layer inputs incorporation.

DPT(V2)	DOSL	PII	PSNR(↑)	SSIM(↑)	LPIPS(↓)
×	×	×	19.86	0.624	0.328
✓	×	×	20.14	0.631	0.321
×	✓	×	20.05	0.627	0.323
×	×	✓	20.08	0.630	0.323
✓	✓	×	20.29	0.642	0.315
×	✓	✓	20.11	0.633	0.319
✓	✓	✓	20.42	0.657	0.312

When all three modules were combined, the model achieved optimal performance across all evaluation metrics, validating the effectiveness and importance of these design choices for sparse view 3D reconstruction.

## Conclusion

This paper presents a novel framework for view synthesis, DASNeRF, which generates detailed new views from minimal input views. Specifically, to address insufficient constraints in few-shot NeRF and the loss of depth details due to missing depth information, we use accurate depth priors combined with local-to-global depth regularization. Additionally, recognizing that few-shot reconstruction relies heavily on effective sampling points for detail reconstruction, we designed a three-layer sampling process, incorporating a dynamic optimal sampling layer on top of the original NeRF sampling. Finally, to address overfitting, we observed that reducing model capacity alleviates overfitting but at the cost of losing details. Therefore, we adopted an MLP structure with per-layer input fusion.Experimental results demonstrate that our method achieves optimal performance on the dataset, successfully achieving accurate 3D reconstruction with richer details.

### Limitation

DASNeRF presents an optimized Neural Radiance Field (NeRF) approach for sparse inputs, specifically aimed at improving sparse reconstruction details. Although DASNeRF has made significant progress in novel view synthesis for small-scale scenes, it still faces challenges when handling large-scale scenes, such as those in the recently released DL3DV-10K large-scale scene dataset [60]. The DL3DV-10K dataset contains multiple complex 3D scenes, featuring a large number of viewpoints and rich geometric details, as well as highly variable depth information and textures. Due to the vast scale of these scenes, which contain numerous dynamic elements and intricate details, the application of DASNeRF in such large-scale environments may encounter bottlenecks in computational resources and memory. Therefore, improving the scalability and detail reconstruction capability of DASNeRF in large scenes remains a key issue for future improvements and research.
